# Effective asymmetric preparation of (*R*)-1-[3-(trifluoromethyl)phenyl]ethanol with recombinant *E. coli* whole cells in an aqueous Tween-20/natural deep eutectic solvent solution

**DOI:** 10.1186/s13568-021-01278-6

**Published:** 2021-08-19

**Authors:** Wenjin Zhuang, Hanyu Liu, Ying Zhang, Junyao He, Pu Wang

**Affiliations:** 1 Key Laboratory of Green Pharmaceutical Technologies and Related Equipment of Ministry of Education, College of Pharmaceutical Science, Zhejiang University of Technology, Hangzhou, 310014 China; 2 Zhejiang Pharmaceutical College, Ningbo, 315100 China

**Keywords:** Biotransformation, Whole-cell catalysis, Surfactant, Natural deep eutectic solvent, Chiral alcohol

## Abstract

(*R*)-1-[3-(Trifluoromethyl)phenyl]ethanol ((*R*)-MTF-PEL) is an important chiral building block for the synthesis of a neuroprotective compound, (*R*)-3-(1-(3-(trifluoromethyl)phenyl)ethoxy)azetidine-1-carboxamide. In this work, an effective whole-cell-catalyzed biotransformation was developed to produce (*R*)-MTF-PEL, and its productivity was increased by medium engineering strategy. The recombinant *E. coli* BL21(DE3)-pET28a(+)*-*LXCAR-S154Y variant affording carbonyl reductase was adopted for the reduction of 3'-(trifluoromethyl)acetophenone to (*R*)-MTF-PEL with enantiomeric excess (ee) > 99.9%. The addition of 0.6% Tween-20 (w/v) boosted the bioreduction, because the substrate concentration was increased by 4.0-fold than that in the neat buffer solution. The biocatalytic efficiency was further enhanced by introducing choline chloride: lysine (ChCl:Lys, molar ratio of 1:1) in the reaction medium, because the product yield reached 91.5% under 200 mM substrate concentration in the established Tween-20/ChCl:Lys-containing system, which is the highest ever reported for (*R*)-MTF-PEL production. The optimal reduction conditions were as follows: 4% (w/v) ChCl:Lys, 12.6 g (DCW)/L recombinant *E. coli* cells, pH 7.0, 30 ℃ and 200 rpm, reaction for 18 h. The combined strategy of surfactant and NADES has great potential in the biocatalytic process and the synthesis of chiral alcohols.

**Supplementary Information:**

The online version contains supplementary material available at 10.1186/s13568-021-01278-6.

## Key points

1. An effective bioprocess for the (*R*)-MTF-PEL preparation was developed.
2. Integration of Tween-20 and ChCl:Lys significantly improved catalytic efficiency.

## Introduction

**Scheme 1:**
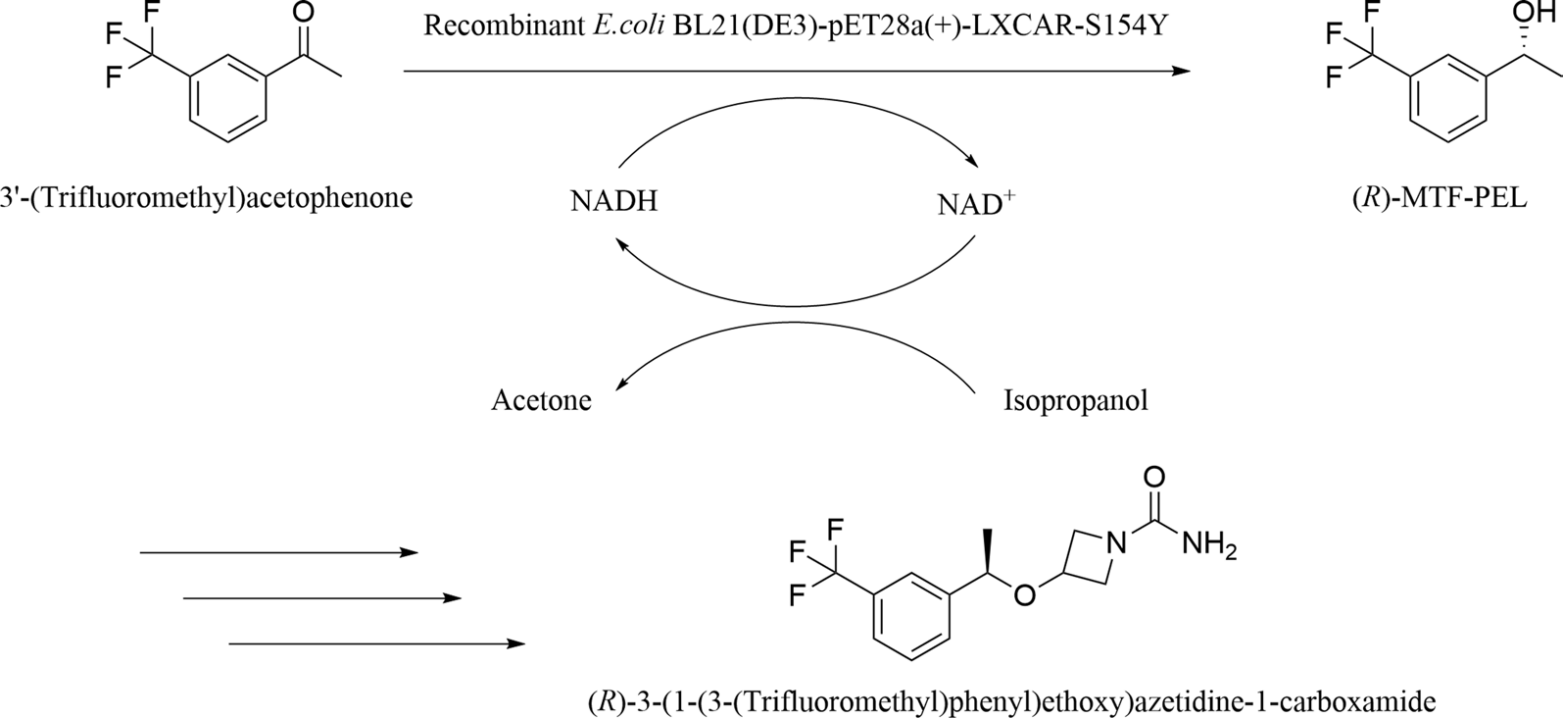
Sketch of the synthesis for (*R*)-MTF-PE and (*R*)-3-(1-(3-(trifluoromethyl)phenyl)ethoxy)azetidine-1-carboxamide

Optically active chiral alcohols play an essential role in medicinal chemistry (Brands et al. 2003; Chen and de Souza 2019; Perrone et al. 2008; Vitale et al. 2020). (*R*)-1-[3-(Trifluoromethyl)phenyl]ethanol ((*R*)-MTF-PEL) is a key chiral building block for the preparation of neuroprotective compounds like (*R*)-3-(1-(3-(trifluoromethyl)phenyl)ethoxy)azetidine-1-carboxamide (Scheme 1) (Snape et al. 2001). A chemical method for (*R*)-MTF-PEL production using [Mn(CO)_2_(**1**)]Br as catalyst was reported, with 99% yield and 97% ee value under 0.5 mM 3'-(trifluoromethyl)acetophenone concentration (Passera and Mezzetti 2020). A metal catalyst was involved in this chemical approach. The carbonyl reductase from *Kluyveromyces thermotolerans* has been reported to reduce 10 mM of 3'-(trifluoromethyl) acetophenone with quantitative conversion and ee > 99% (Xu et al. 2012). Whole-cell biocatalysis is more attractive due to high stereoselectivity and good application potential, with mild reaction conditions and no need for expensive coenzymes (De Carvalho 2017; Domínguez and Kohlmann 2012; Goldberg et al. 2007). *Microbacterium oxydans* C3 has been applied in the biosynthesis of (*R*)-MTF-PEL with 79% conversion under 5 mM 3'-(trifluoromethyl) acetophenone concentration (Gai et al. 2013). For (*R*)-MTF-PEL production, the biocatalytic efficiency is still unsatisfactory. Thus, it is necessary to develop a more efficient whole-cell biotransformation process for its production at high substrate concentration.

The low water solubility and mass transfer of hydrophobic synthetic substrates limit the efficiency of biocatalytic process (Oda 2020; Pollard et al. 2006; Tang et al. 2020). These limitations can be alleviated to a certain extent by introducing proper surfactant into the reaction medium. The effect of surfactants in the biotransformation process might be attributed to their ability to form micellar systems with hydrophobic substrates (Cortes-Clerget et al. 2019; Elsayed et al. 2019; Santos et al. 2016). The hydrophobic compound is distributed to the hydrophobic center of the surfactant micelle, thus the concentration of the substrate/product in the aqueous solution drops below inhibitory content, consequently increasing the water solubility of hydrophobic compound (Su et al. 2010). In the process for (*S*)-(4-chlorophenyl)-(pyridin-2-yl)methanol ((*S*)-CPMA) production catalyzed by *Kluyveromyces* sp*.* CCTCC M2011385 whole-cell, the substrate concentration was increased to 6 g/L in a system consisted of PEG4000 compared with 2 g/L in the pure water system (Ni et al. 2012). In the enzymatic process of ginsenoside Rh_2_, the conversion of Rg_3_ was improved 25% in the surfactant-containing system than that in the medium without surfactant. Besides, surfactants exhibit a beneficial effect on the stability of enzyme, and some biocompatible nonionic surfactants can be applied in this enzymatic reaction (Su et al. 2010).

Natural deep eutectic solvents (NADESs), a class of emerging non-conventional solvents, have been employed in many fields due to their advantages including environmentally friendly, easy to prepare and good biocompatibility (Hansen et al. 2021). In the process of biocatalytic reactions, it has been reported that NADESs have some functions such as improving cell membrane permeability, facilitating the mass transport and alleviating substrate inhibition (Cicco et al. 2021; Gotor-Fernandez and Paul 2019; Panic et al. 2021; Patzold et al. 2019; Perna et al. 2020). NADES was typically composed of a hydrogen bond acceptor (HBA, such as choline chloride (ChCl)), and a hydrogen bond donor (HBD, such as polyols, carboxylic acids, amino acids, or sugars) (Benvenutti et al. 2019). *Kurthia gibsonii* SC0312 catalyzed the bioreduction of 2-hydroxyacetophenone to (*R*)-1-phenyl-1,2-ethanediol in 2–16% (v/v) choline chloride:1,4-butanediol (ChCl:Bd, 1:1)-containing medium, giving a 80% yield and optical purity > 99%. ChCl:Bd in this reaction appropriately improved the permeability of cell membrane (Peng et al. 2020). During the reduction of ethyl 4-chloro-3-oxobutanoate (COBE) by recombinant *E. coli* CCZU-T15 in 12.5% (v/v) choline chloride/glycerol-water media containing 7.5 mM Tween-80, the product (*S*)-4-chloro-3-hydroxybutyrate ((*S*)-CHBE) was obtained in nearly quantitative yield under 1000 mM COBE. The cellular membrane became more permeabilized and its integrity was destroyed with DES and surfactant (Dai et al. 2017). It exhibited that surfactant along with NADES in reaction medium can effectively promote the whole-cell biocatalytic process, and manifested applied potential in the biotransformation.

In this work, the recombinant *E. coli* BL21(DE3)-pET28a(+)*-*LXCAR-S154Y variant turned out to be a suitable biocatalyst for synthesizing (*R*)-MTF-PEL after the strain screening, and the critical factors affecting the bioreductive process were investigated. To further enhance the catalytic efficiency, surfactant was introduced in the reaction medium with higher substrate loading. Moreover, a surfactant/NADES-containing system was constructed, and the bioreductive conditions were optimized. Furthermore, the effects of different surfactants and NADESs on the substrate solubility and cell membrane permeability were explored. This study paved a way for the efficient production of (*R*)-MTF-PEL.

## Materials and methods

### Materials

3'-(Trifluoromethyl)acetophenone (purity > 97%) was acquired from Aladdin Chemistry Co., Ltd. (Shanghai, China). (*R*)-MTF-PEL (purity > 98%) was obtained by Daicel Chiral Technologies (China) Co., Ltd. Strain *Cyberlindnera saturnus* ZJPH1807 (CCTCC M 2019215), *Galactomyces geotrichum* ZJPH1810 (CCTCC M 2019822), *Geotrichum candidum* ZJPH1704 (CCTCC M 2017380) and *Pseudomonas aeruginosa* ZJPH1504 (CCTCC M 2016188) were isolated from soil samples in our previous screening and preserved in our laboratory. The *Escherichia coli* BL21(DE3)-pET28a(+)-LXCAR-S154Y variant was acquired in our previous study (Wang et al. 2014). NADESs used in the experiments were synthesized and supplied by Shanghai Chengjie Chemical Co. Ltd (Shanghai, China). The ^1^H NMR spectrum of ChCl:Lys (1:1) was shown in Additional file 1: Fig. S3. Other chemicals used in the experiments were commercially available with analytical grade.

### Microorganisms and cultivation conditions

Wild type microorganisms for (*R*)-MTF-PEL synthesis were screened from soil samples that were from different regions of China, including Shandong, Henan, Anhui, and Zhejiang province. A substrate-directed screening approach was employed referring to our previous work (Sun et al. 2016). The enriched medium for the required strains contained the following components (g/L): 3'-(trifluoromethyl)acetophenone 2, NaCl 0.5, KH_2_PO_4_ 1, MgSO_4_·7H_2_O 0.5, (NH_4_)_2_SO_4_ 2, with the initial pH of 6.5. The culture was then inoculated into the isolation medium containing (g/L): glucose 15, yeast extract 5, peptone 5, K_2_HPO_4_ 0.5, KH_2_PO_4_ 0.5, MgSO_4_·7H_2_O 0.5, NaCl 1, (NH_4_)_2_SO_4_ 1, with the initial pH of 6.5. After the incubation and streaking of the single colony continuously on the isolation medium agar, various isolates were acquired and used for the bioreduction of 3'-(trifluoromethyl)acetophenone. The recombinant *E. coli* BL21(DE3)-pET28a(+)*-*LXCAR-S154Y was acquired in our previous work, which is a recombinant carbonyl reductase variant (LXCAR-S154Y) derived from *Leifsonia xyli* HS0904. The cultivation conditions of the recombinant *E. coli* variant were similar to the literature (Wang et al. 2014). After the cultivation, the incubated cells were collected by the centrifugation at 4 ℃ and 9000 rpm, and then used for the 3'-(trifluoromethyl)acetophenone bioreduction. Furthermore, some isolated strains stored in our laboratories were also cultivated by shaken culture and evaluated their abilities for the bioreduction of 3'-(trifluoromethyl)acetophenone.

### Enzymatic activity assay

Enzyme activity was assessed by detecting the absorbance variation at 340 nm of NAD(P)H. For cell-free extract, the cells were resuspended in PBS buffer (200 mM, pH 7.5), and disrupted by ultrasonication (500 W, work 2 s, stop 5 s) in an ice-bath for 30 min. Then, the lysed cells were subjected to centrifugation at 12,000 rpm for 10 min to remove the cell debris, and the supernatant was collected as cell free extract. Each reduction reaction was performed in the reaction mixture containing 1.0 mL PBS buffer (200 mM, pH 7.5), 1.0 mL cell free extract, supplemented with various additives or not, 0.3 mM NAD^+^, 200 mM 3'-(trifluoromethyl)acetophenone. One unit (U) of carbonyl reductase activity is expressed as producing or consuming 1 µmol of NAD(P)H per minute.

### The general bioreductive process of 3'-(trifluoromethyl)acetophenone to (*R*)-MTF-PEL

The bioreduction of 3'-(trifluoromethyl)acetophenone was undertaken at 30 °C and 200 rpm in 50 mL Erlenmeyer flask, which containing a certain amount of 3'-(trifluoromethyl)acetophenone, co-substrate and microbial whole cells, either in the PBS buffer system or in a surfactant and NADES containing medium. After the completion of the bioreduction, the reaction mixture was extracted twice with an equal volume of EtOAc, and the resultant organic phase was subjected to gas chromatography (GC) analysis. Each trial was performed in triplicate. Details about 3'-(trifluoromethyl)acetophenone concentration, co-substrate concentration, surfactant and NADES content, buffer pH, reaction temperature, cell concentration and reaction time were specified for each case.

### Measurement of substrate solubility

The substrate water solubility was determined according to the method similar to the previous report (Wang et al. 2018). Excess 3'-(trifluoromethyl)acetophenone was dispersed in a buffer solution containing certain amount of surfactant and NADES or not, and mixed adequately at 30 °C and 200 rpm for 24 h. The obtained samples were then centrifuged under 12,000 rpm for 5 min. The resultant supernatant was extracted twice with an equal volume of EtOAc. The obtained organic phase was then determined by GC.

### Analytical method

The concentrations of the residual 3'-(trifluoromethyl)acetophenone and the resultant (*R*)-MTF-PEL were quantified by GC (Agilent GC 7820A) with a chiral CP-Chirasil-Dex CB column. Injector and detector temperatures were set as 250 ℃. The initial column temperature was 115 ℃, kept for 2 min, and then increased to 140 ℃ at a rate of 3 ℃/min. The retention times of 3'-(trifluoromethyl)acetophenone, *n*-dodecane, (*R*)-MTF-PEL and (*S*)-MTF-PEL were 2.64 min, 3.94 min, 6.35 min and 6.92 min, respectively (Additional file 1: Fig. S1). The ^1^H NMR and ^13^C NMR spectra of the product were also determined (Additional file 1: Fig. S2).

To calculate the product yield and ee value, the calibration curves of 3'-(trifluoromethyl)acetophenone and (*R*)-MTF-PEL were firstly established using the corresponding standard of substrate or product with *n*-dodecane as an internal standard (Additional file 1: Fig. S5).

The product yield was calculated as follows:

$${\text{Yield }}\left( \% \right) \, = {\text{ C}}_{P} /{\text{C}}_{0} \times {1}00\%$$

C_*p*_ and C_*0*_ are the concentrations of the resultant (*R*)-MTF-PEL and initial concentration of 3'-(trifluoromethyl)acetophenone, respectively.

The ee value was calculated as follows:

$${\text{ee }} = \, ({\text{C}}_{R} - {\text{C}}_{S} )/({\text{C}}_{R} + {\text{C}}_{S} ) \, \times {1}00\%$$

C_*R*_ and C_*S*_ are the concentrations of (*R*)-MTF-PEL and (*S*)-MTF-PEL, respectively.

### Cell metabolic activity assay

The metabolic activity retention (MAR) value of recombinant *E. coli* cells was assessed by the method recorded in our previous research (Xia et al. 2020). The cells were pre-incubated for 6 h at 30 ºC and 200 rpm in a buffer solution containing surfactant or NADES, with 3'-(trifluoromethyl)acetophenone or not. After that, 10 g/L glucose solution was supplemented and further incubated for 4 h. The glucose concentration in various systems was monitored with the biological sensing analyzer (SBA-90).

### Assess of cell membrane permeability

The cellular membrane permeability was appraised by the variation of OD values at 260 nm and 280 nm respectively. The cells were incubated at 30 ºC, 200 rpm in various surfactant and NADES containing systems. After 6 h incubation, cells were discarded by centrifugation, and the absorbance of obtained supernatants containing NADESs or not was measured at 260 nm and 280 nm by a microplate reader (SpectraMAX M5). Cell membrane permeability was also assessed by the method of scanning electron microscope (SEM) images of the cells pretreated by surfactant or NADES with Hitachi SU-8010 (SEM) observation.

## Results

### Screening of microorganisms for 3'-(trifluoromethyl)acetophenone biotransformation

As shown in Table 1, several strains were investigated for their abilities to synthesize (*R*)-MTF-PEL. Most of them reduced 3'-(trifluoromethyl)acetophenone to (*S*)-isomer, while the recombinant *E. coli* BL21(DE3)-pET28a(+)-LXCAR-S154Y showed the performance to produce (*R*)-MTF-PEL with > 99.9% enantioselectivity. Thus, this strain was determined for the subsequent experiments.

**Table 1:** Screening of biocatalysts

Strain	Yield (%)	ee (%)	Configuration
Recombinant *E. coli* BL21(DE3)-pET28a(+)*-*LXCAR-S154Y^a^	26.9	> 99.9	(*R*)
*Cyberlindneras saturnus* ZJPH1807 (CCTCC M 2019215)	35.4	92.9	(*S*)
*Galactomyces geotrichum* ZJPH1810 (CCTCC M 2019822)	32.7	87.5	(*S*)
*Geotrichum candidum* ZJPH1704 (CCTCC M 2017380)	48.6	89.9	(*S*)
*Pseudomonas aeruginosa* ZJPH1504 (CCTCC M 2016188)	15.6	66.7	(*S*)
AH-18-3^b^	47.4	99.9	(*S*)
GS-19-5^b^	32.1	99.9	(*S*)
SX-18-1^b^	37.6	98.2	(*S*)
SD-18-6^b^	35.5	92.2	(*S*)
HN-17-1^b^	40.8	89.1	(*S*)

^a^Recombinant *E. coli* BL21(DE3)-pET28a(+)-LXCAR-S154Y affording carbonyl reductase (LXCAR) gene from *Leifsonia xyli* HS0904 was acquired in our previous study (Wang et al. 2014)

^b^Isolates were screened out from different soil samples

### Effect of co-substrate on (*R*)-MTF-PEL production in buffer reaction medium

**Fig. 1:**
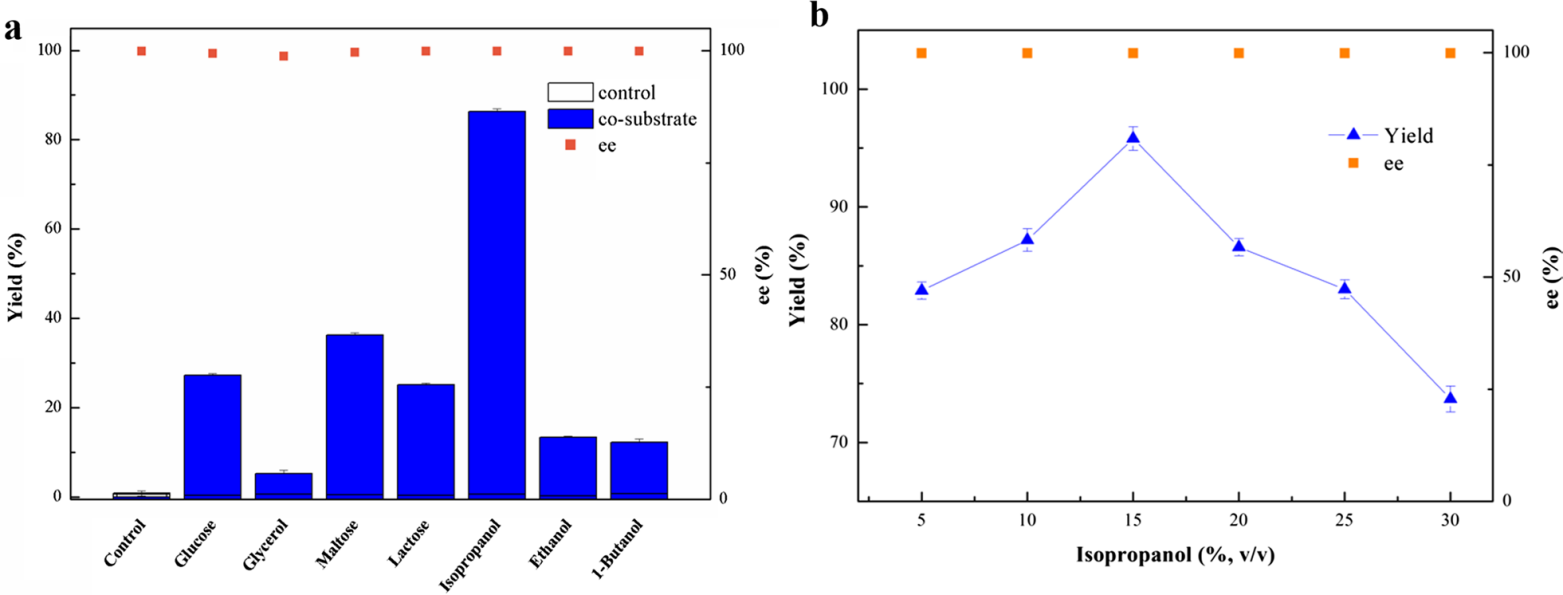
Effects of different co-substrate (**a**) and various isopropanol proportion (**b**) on the (*R*)-MTF-PEL production with recombinant *E. coli* cells in PBS buffer medium. Reaction conditions: 50 mM 3'-(trifluoromethyl)acetophenone (**a, b**), 10% (w/v) different co-substrate (**a**), 10.57 g (DCW)/L recombinant *E. coli* cells, PBS buffer (pH 7.0) (**a, b**), 30 ℃, 200 rpm, 12 h

Co-substrate plays a critical role in the biocatalytic reduction process catalyzed with whole cells. It can promote cofactor regeneration, thus no need of costly cofactors addition (Goldberg et al. 2007; Sharma et al. 2017). During the biosynthesis of (*R*)-MTF-PEL, seven co-substrates were added separately in the buffer medium to evaluate their performances in the bioreduction. As shown in Fig. 1a, only 0.8% yield was obtained in the buffer solution without co-substrate addition, while all co-substrates improved the yield. Among them, isopropanol as co-substrate gave the best performance, with 86.2% yield at 50 mM substrate concentration. Subsequently, the proportion of isopropanol was further optimized in the range of 5–30% (v/v). The highest yield of 95.8% was achieved with the supplement of 15% (v/v) isopropanol as co-substrate (Fig. 1b), which is nearly 119.8-fold increase compared to that of no addition of co-substrate. Further increase in isopropanol proportion leads to the drops in product yield. It is probably due to the fact that excessive isopropanol manifested toxic to the microbial cells (Sharma et al. 2017).

### Effects of key reaction parameters on (*R*)-MTF-PEL production in buffer reaction medium

**Fig. 2:**
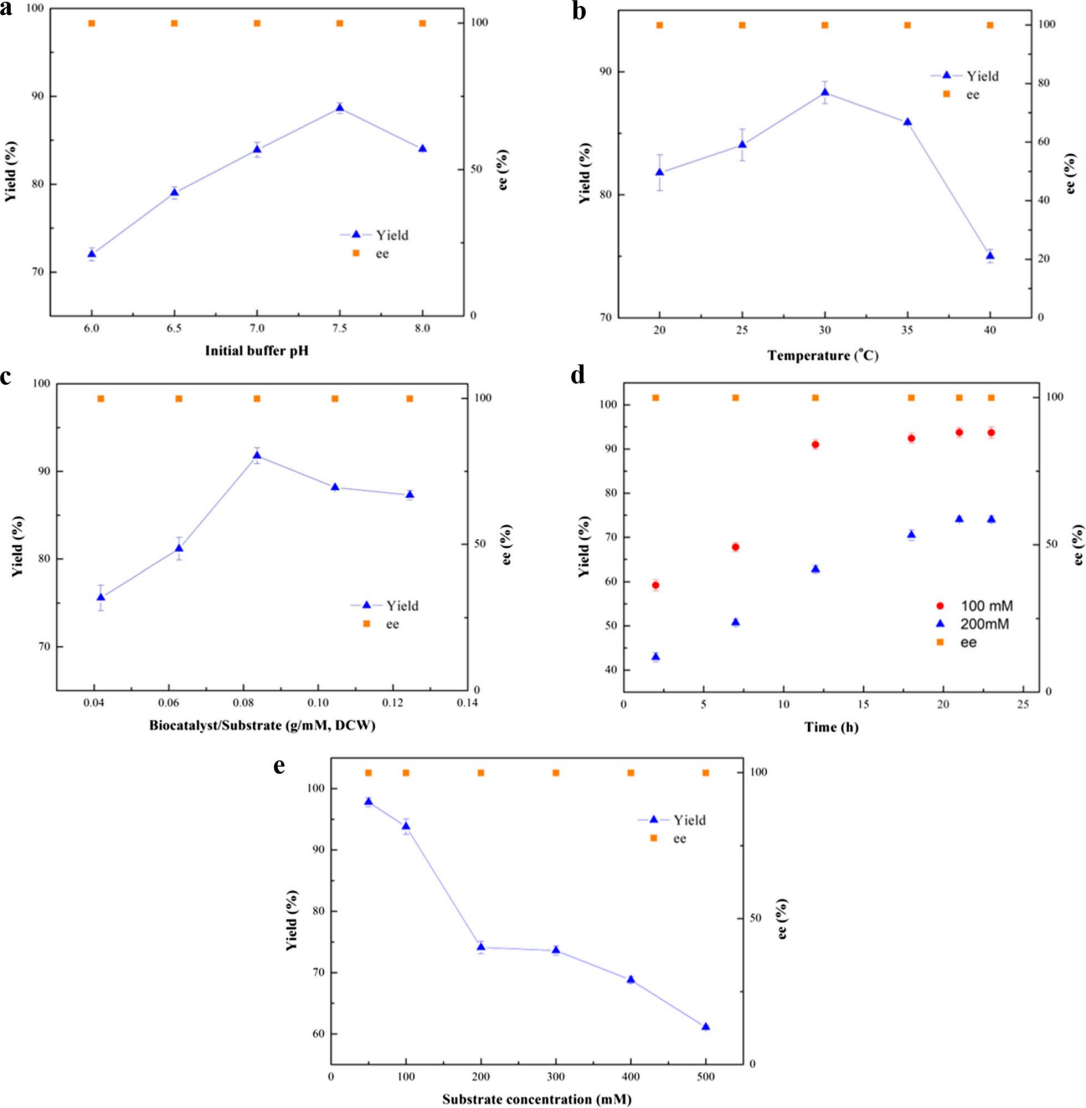
Effects of key reaction parameters on the (*R*)-MTF-PEL production with recombinant *E. coli* cells in PBS buffer medium. Reaction conditions: **a** Effect of initial buffer pH: 100 mM 3'-(trifluoromethyl)acetophenone, 15% (v/v) isopropanol, 10.57 g (DCW)/L recombinant *E. coli* cells, 30 ℃, PBS buffer, 200 rpm, 12 h. **b** Effect of temperature: 100 mM 3'-(trifluoromethyl)acetophenone, 15% (v/v) isopropanol, 10.57 g (DCW)/L recombinant *E. coli* cells, PBS buffer (pH 7.5), 200 rpm, 12 h. **c** Effect of the ratio of biocatalyst to substrate concentration: 100 mM 3'-(trifluoromethyl)acetophenone, 15% (v/v) isopropanol, various recombinant *E. coli* cells as biocatalyst, PBS buffer (pH 7.5), 30 ℃, 200 rpm, 12 h. **d** Time course of (*R*)-MTF-PEL production with recombinant *E. coli* cells in PBS buffer medium: 15% (v/v) isopropanol, PBS buffer (pH 7.5), 30 ℃, 200 rpm, 8.6 g (DCW) /L recombinant *E. coli* cells under 100 mM substrate concentration (Black circle), 17.6 g (DCW) /L recombinant *E. coli* cells under 200 mM substrate concentration (Black up-pointing triangle). **e** Effect of substrate concentration on the (*R*)-MTF-PEL production with recombinant *E. coli* cells: 15% (v/v) isopropanol, 0.086 g (DCW)/mM recombinant *E. coli* cells, PBS buffer (pH 7.5), 30 ℃, 200 rpm, 21 h

The effect of buffer pH (from 6.0 to 8.0) on the asymmetric reduction was examined to determine a suitable level. As shown in Fig. 2a, the maximum yield of (*R*)-MTF-PEL was obtained at pH 7.5. The effect of temperature on the product yield was shown in Fig. 2b. In the range of 20 to 40 °C, the yield increased with the temperature elevating from 20 to 30 °C, and then decreased at the temperature above 30 °C. During the asymmetric biological reduction, the biocatalyst dosage is a significant issue, which can affect the efficiency of biocatalytic reaction (Wu et al. 2020). As shown in Fig. 2c, 8.6 g (DCW) /L recombinant *E. coli* cells under 100 mM substrate concentration is preferred for this reaction, while 17.6 g (DCW) /L recombinant *E. coli* cells under 200 mM substrate loading. Figure 2d showed that under the optimal reaction conditions of pH 7.5, 30 °C, 17.6 g (DCW) /L recombinant *E. coli* cells under 200 mM substrate concentration, reaction for 21 h, 93.8% yield was acquired under 100 mM substrate concentration, and 74.1% yield at 200 mM substrate loading, respectively.

It was found that the product yield decreased noticeably with the increasing substrate concentration (Fig. 2e), which might be attributed to the toxicity of substrate and/or product to the microbial cells. Thus, relieving the inhibition from substrate and product was supposed to enhance the efficiency of the bioprocess.

### Effect of surfactants on the (*R*)-MTF-PEL synthesis

**Fig. 3:**
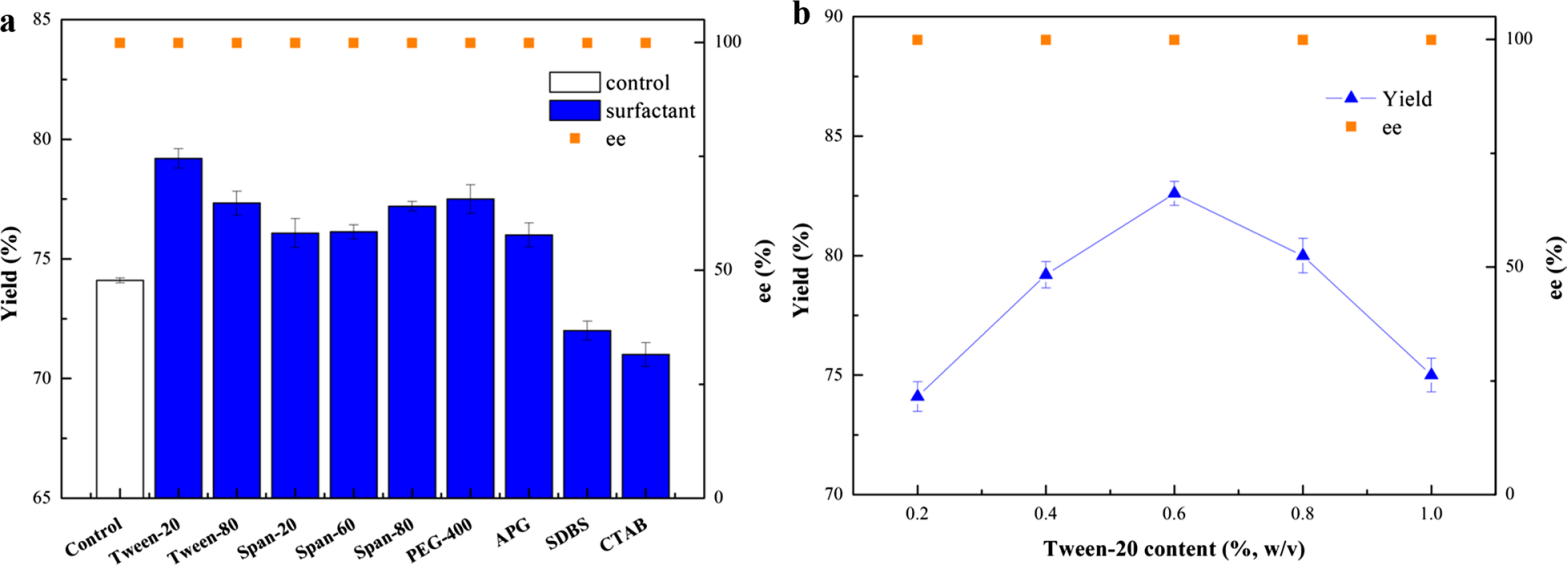
Effect of different surfactant (**a**), and various Tween-20 content (**b**) on the (*R*)-MTF-PEL production with recombinant *E. coli* cells. Reaction conditions: 200 mM 3'-(trifluoromethyl)acetophenone, 15% (v/v) isopropanol, 17.2 g (DCW)/L recombinant *E. coli* cells, PBS buffer (pH 7.5) (**a, b**), 0.4% (w/v) various surfactants (**a**), 30 ℃, 200 rpm, 21 h

In general, the low solubility of hydrophobic substrates decreased the biocatalytic yield owing to the mass-transfer hindrance (Sheldon and Pereira 2017). The solubility of 3'-(trifluoromethyl)acetophenone was 425.5 mg/L in PBS buffer solution (Additional file 1: Table S1), which hindered the efficient production of (*R*)-MTF-PEL. To improve the substrate solubility in aqueous buffer system and increase the reaction efficiency, nine surfactants were introduced individually to the reaction medium and their performances were evaluated (Fig. 3a). Among the examined nine surfactants, except cetyltrimethyl ammonium bromide (CTAB) and sodium dodecyl benzene sulfonate (SDBS) were ionic surfactants, the other seven were non-ionic surfactants. It was noticed that all non-ionic surfactants enhanced the biocatalytic efficiency effectively. Among them, Tween-20 gave the best performance and the product yield achieved 79.2% under 200 mM substrate concentration. The effect of Tween-20 with a proportion content of 2 to 10 g/L on the biocatalytic reduction was subsequently investigated, and the results are given in Fig. 3b. At 0.6% (w/v) Tween-20, the product yield reached 82.6%, comparatively 74.1% yield in the neat buffer system. Further increase of Tween-20 content resulted in an apparent drop in product yield. The optimal concentration of Tween-20 is 0.6% (w/v).

To understand the influence of Tween-20 on the reaction, the solubility of 3'-(trifluoromethyl)acetophenone in different media was further examined. As displayed in Additional file 1: Table S1, the solubility of 3'-(trifluoromethyl)acetophenone was 739.6 mg/L in a Tween-20-containing reaction medium, which was increased by 1.7-fold compared to Tween-20-free reaction system. These results implied that the enhanced solubility of substrate in the Tween-20-containing system is accounted for the increase in product yield.

### Effect of NADESs on the (*R*)-MTF-PEL synthesis

**Fig. 4:**
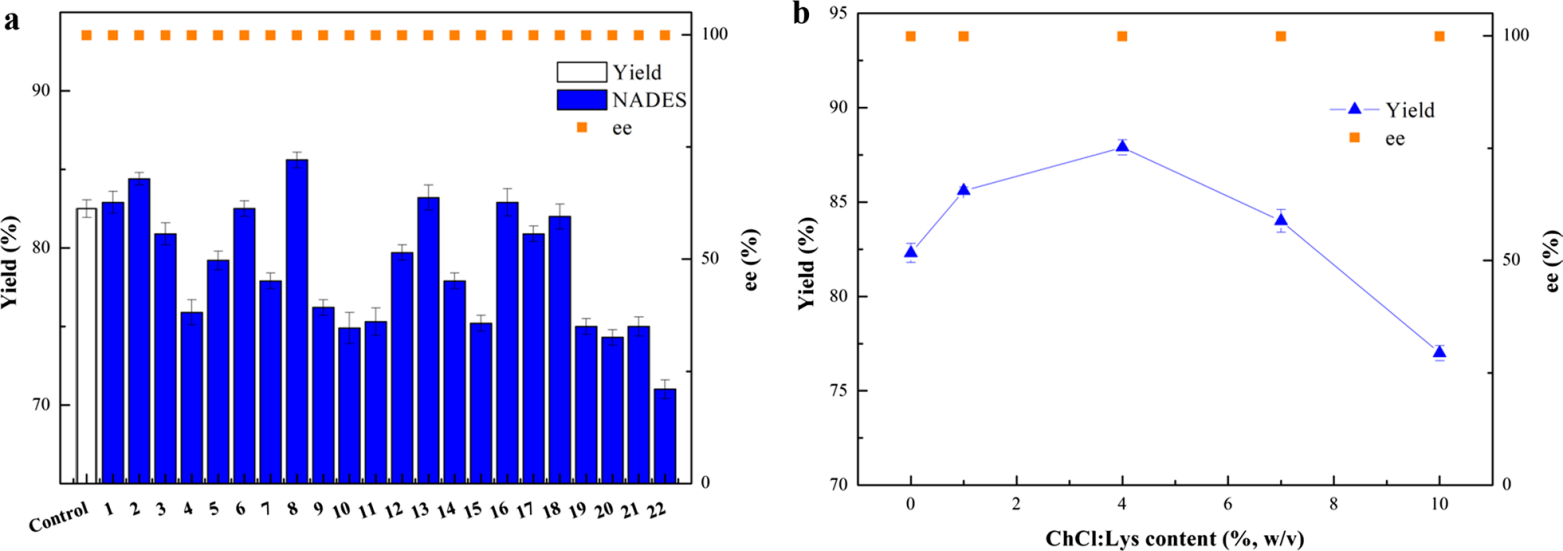
Effect of various NADESs (**a**) and different ChCl:Lys content (**b**) on the (*R*)-MTF-PEL production with recombinant *E. coli* cells. Reaction conditions: 200 mM 3'-(trifluoromethyl)acetophenone, 15% (v/v) isopropanol, 0.6% (w/v) Tween-20, 17.2 g (DCW)/L recombinant *E. coli* cells, PBS buffer (pH 7.5) (**a, b**), 1% (w/v) various NADESs (**a**), 30 ℃, 200 rpm, 21 h; The number of various NADESs is listed in Table 2

Biocatalytic reactions might be inhibited by the low mass-transfer efficiency and the toxicity of hydrophobic substrates/products toward the biocatalyst (Erol and Hollmann 2021; Jung et al. 2013). It was reported that NADESs can improve the mass transfer efficiency by enhancing the permeability of cell membrane, and alleviate substrate/product inhibition (Xia et al. 2020). In this work, twenty-two types of NADESs were introduced individually into the reaction medium to further improve the conversion efficiency. The NADESs investigated in the present study are consisted of three different types of HBAs (ChCl, betaine, *L*-proline), and various HBDs (amino acids, sugars, and alcohols etc.) (Table 2). As depicted in Fig. 4a, the product yields in ChCl-based NADES-containing system were generally higher than the others. Among the ChCl-based NADESs, ChCl:Lys (1:1) exerted the best impact on the product yield. As shown in Fig. 4b, when the content of ChCl:Lys (1:1) is 4% (w/v), the maximum product yield of 87.9% was gained. So, the appropriate ChCl:Lys (1:1) content was 4% (w/v).

**Table 2:** NADESs and their mole ratio

Entries	NADESs	Abbreviation of NADESs	Mole ratio (HBA:HBD)
1	Choline chloride: glycine	ChCl:Gly	1:1
2	Choline chloride: glutamate	ChCl:Glu	1:1
3	Choline chloride: alanine	ChCl:Ala	1:1
4	Choline chloride: tryptophan	ChCl:Trp	1:1
5	Choline chloride: tyrosine	ChCl:Tyr	1:1
6	Choline chloride: glutathione	ChCl:GSH	1:1
7	Choline chloride: cysteine	ChCl:Cys	1:1
8	Choline chloride: lysine	ChCl:Lys	1:1
9	Choline chloride: lysine	ChCl:Lys	1:2
10	Choline chloride: lysine	ChCl:Lys	2:1
11	Choline chloride: mycose	ChCl:Myc	1:1
12	Choline chloride: fructose	ChCl:Fru	1:1
13	Choline chloride: ethylene glycol	ChCl:EG	1:1
14	Choline chloride: urea	ChCl:U	1:1
15	Choline chloride: urea	ChCl:U	1:2
16	Choline chloride: urea	ChCl:U	2:1
17	Choline chloride: isopropanol	ChCl:IPA	1:1
18	Choline chloride: isopropanol	ChCl:IPA	1:2
19	Betaine: lysine	B:Lys	1:2
20	Betaine: isopropanol	B:IPA	1:1
21	*L*-proline: lysine	P:Lys	1:1
22	*L*-proline: isopropanol	P:IPA	1:1

To investigate the effect of different additives on coenzyme regeneration, isopropanol, Tween-20, ChCl:Lys (1:1) and NAD^+^ were added respectively in the cell-free extract of recombinant *E. coli* cells to determine the specific enzyme activity by detecting the changes in absorption at 340 nm. As shown in Table 3, the addition of ChCl:Lys (1:1) in the cell-free extract of recombinant *E. coli* cells can efficiently boost NADH regeneration, which confirmed that ChCl:Lys (1:1) plays a role in promoting cofactor regeneration during 3'-(trifluoromethyl)acetophenone bioreduction catalyzed by recombinant *E. coli* cells.

**Table 3:** Redox specific activities of cell-free extract on different additives and NAD^+^

Isopropanol^a^	Tween-20^b^	ChCl:Lys^c^	NAD^+^	Specific activity (U/mg)
−	−	−	−	ND
−	−	−	+	ND
+	−	−	−	ND
+	−	−	+	2.21 ± 0.04
−	+	−	+	ND
−	−	+	−	ND
−	−	+	+	1.73 ± 0.04
+	−	+	+	3.07 ± 0.09

Reaction conditions: 1.0 mL phosphate buffer (pH 7.5), 1.0 mL cell-free extract (5.25 mg of total protein), with or without various additives, 0.3 mM NAD^+^

“ + ” means added the additives; “−”means not added; “ND” means not detected

^a^15% (v/v) isopropanol

^b^0.6% (w/v) Tween-20

^c^4% (w/v) ChCl:Lys (1:1)

Subsequently, the asymmetric reduction performance of ChCl:Lys (1:1) was compared with the individual addition of ChCl, lysine, or the mixture of individual components (ChCl + lysine) with the same molar ratio to ChCl:Lys (1:1) (Additional file 1: Table S3). 85.6% product yield was obtained in reaction system contained 4% (w/v) ChCl:Lys (1:1). However, 80.8% yield was produced in the bioreduction system containing the mixture of ChCl and lysine. The results exhibited that ChCl:Lys (1:1) as a HBA-HBD complex may play an important role in asymmetric reduction with 4% (w/v) content, instead of being dissociated into separated components. Similar results have been reported by Mao et al. In the 1,2-dehydrogeneration of cortisone acetate (CA) by whole cells of *Arthrobacter simplex*, the catalytic performance in the 6% ChCl:urea-containing system was better than those in their individual components (ChCl or urea) and their mixture (Mao et al. 2018).

To further investigate the combined effect of Tween-20 and ChCl:Lys on the reaction, the asymmetric reduction of 3'-(trifluoromethyl)acetophenone were conducted in different reaction system, and the comparison of corresponding product yield was illustrated in Additional file 1: Table S2. It was found that the best product yield was acquired in the Tween-20/ChCl:Lys-containing system. The results indicated that the simultaneous addition of Tween-20 and ChCl:Lys in the reaction system displayed a synergistic effect on the biocatalytic reduction.

### Effects of important reductive parameters on the (*R*)-MTF-PEL production in the Tween-20/ChCl:Lys-containing reaction medium

**Fig. 5:**
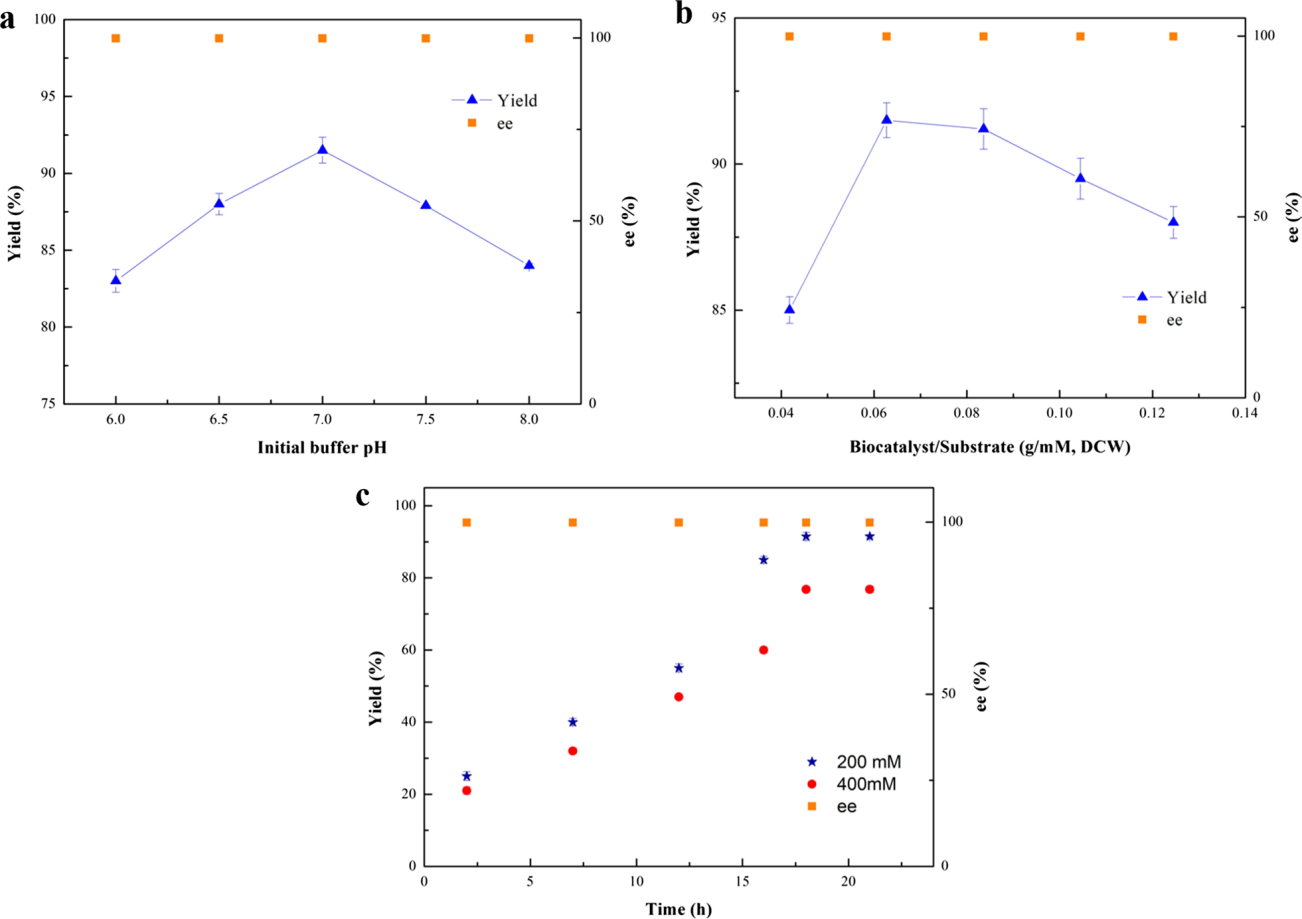
Effects of key reaction parameters on the (*R*)-MTF-PEL production with recombinant *E. coli* cells in a Tween-20/ChCl:Lys-containing system. Reaction conditions: **a** Effect of initial buffer pH: 200 mM 3'-(trifluoromethyl)acetophenone, 15% (v/v) isopropanol, 0.6% (w/v) Tween-20, 4% (w/v) ChCl:Lys, 17.2 g (DCW)/L recombinant *E. coli* cells, 30 ℃, 200 rpm, 21 h. **b** Impact of the ratio of biocatalyst/substrate: 200 mM 3'-(trifluoromethyl)acetophenone, 15% (v/v) isopropanol, 0.6% (w/v) Tween-20, 4% (w/v) ChCl:Lys, PBS buffer (pH 7.0), 30 ℃, 200 rpm, 21 h. **c** Time course of (*R*)-MTF-PEL production under 200 mM and 400 mM substrate concentration: 15% (v/v) isopropanol, 0.6% (w/v) Tween-20, 4% (w/v) ChCl:Lys, PBS buffer (pH 7.0), 30 ℃, 200 rpm, 21 h. 12.6 g (DCW)/L *E. coli* cells under 200 mM substrate concentration (Black star), 25.2 g (DCW)/L recombinant *E. coli* cells under 400 mM substrate concentration (Black circle)

The effects of buffer pH and the biocatalyst dosage on the bioreduction in the Tween-20/ChCl:Lys-containing system were subsequently investigated. As shown in Fig. 5a, the optimum buffer pH varied from 7.5 in neat PBS buffer medium to 7.0 in the Tween-20/ChCl:Lys-containing system, which probably due to the fact that lysine belongs to basic amino acid. As shown in Fig. 5b, the biocatalyst dosage was dropped to 12.6 g (DCW)/L (while 17.6 g (DCW) /L in neat PBS buffer medium), which means less biocatalyst dosage needed in the developed reaction system, thereby the biocatalytic process was more cost-effective. At the optimum conditions, the product yield reached 91.5% under 200 mM substrate concentration, which was 10.8% higher than that in Tween-20-containing system. And the reaction time was shortened from 21 to 18 h (Fig. 5c). The yield achieved 76.8% under 400 mM substrate concentration.

To evaluate the applicability of the established Tween-20/ChCl:Lys-containing system for other ketones reduction reactions, various ketones to their corresponding (*R*)-alcohols in the Tween-20/ChCl:Lys-containing system were investigated (Table 4). It was found that the yields of all tested aromatic ketones were enhanced in the Tween-20/ChCl:Lys-containing system. The results described herein showed that the established Tween-20/ChCl:Lys-containing system is an alternative reaction medium not only for the bioreduction of ketoesters, but also for other aromatic ketones.

**Table 4:** Recombinant *E. coli* cell-catalyzed bioreduction of various ketones in Tween-20/ChCl:Lys-containing system

Substrate	Product	Substrate concentration (mM)	Reaction system	Yield (%)	ee (%)
1-(4-Fluorophenyl)ethanone	(*R*)-1-(4-Fluorophenyl)ethanol	100	PBS buffer	42.2	> 99.9
			Tween-20/ChCl:Lys-containing system	87.0	> 99.9
1-[4-(Trifluoromethyl)phenyl]ethanone	(*R*)-1-[4-(Trifluoromethyl)phenyl]ethanol	100	PBS buffer	88.5	> 99.9
			Tween-20/ChCl:Lys-containing system	95.8	> 99.9
Ethyl acetoacetate	Ethyl (*R*)-3-hydroxybutyrate	1000	PBS buffer	63.2	> 99.9
			Tween-20/ChCl:Lys-containing system	76.4	> 99.9
3,5-Bis(trifluoromethyl)acetophenone	(*R*)-[3,5-Bis(trifluoromethyl)phenyl]ethanol	1000	PBS buffer	69.1	> 99.9
			Tween-20/ChCl:Lys-containing system	86.3	> 99.9

Reaction conditions: 0.063 g (DCW)/mM *E. coli* cells, 15% (v/v) isopropanol, 0.6% (w/v) Tween-20, 4% (w/v) ChCl:Lys, 30 ºC and 200 rpm, reaction for 18 h

### Biocompatibility of various surfactants and NADESs with recombinant *E. coli* cells

**Fig. 6:**
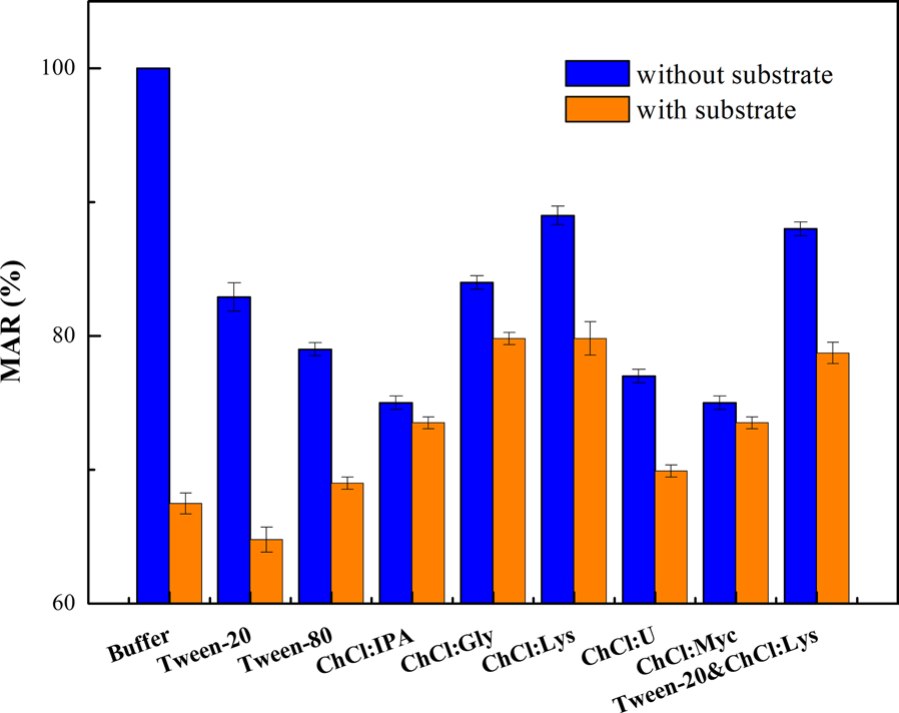
MAR of recombinant *E. coli* cells in different reaction media. Reaction conditions: 200 mM of 3'-(trifluoromethyl)acetophenone, 17.2 g (DCW)/L recombinant *E. coli* cells, 0.6% (w/v) surfactant, 4% (w/v) NADES, PBS buffer (pH 7.0), 3  ℃, 200 rpm

To assess the biocompatibility of various co-solvents in this medium to the biocatalyst, glucose metabolic activity retention (MAR) of the recombinant *E. coli* cells was measured either with substrate or not. As depicted in Fig. 6, the MAR values detected in substrate-free buffer solution were higher than those supplemented with substrate, thus proved that 3'-(trifluoromethyl)acetophenone exerts cytotoxic towards the recombinant *E. coli* cells. Besides, the cells in the surfactant-containing system had comparatively lower MAR than those in aqueous system, which means surfactant exhibited certain toxicity to the cells. Among all tested NADESs, ChCl:Lys showed the best biocompatibility towards the cells with the highest MAR, which demonstrated that ChCl:Lys can weaken the cytotoxicity of the substrate to the microbial cells. In the Tween-20/ChCl:Lys-containing system, the MAR values is lower slightly than that in ChCl:Lys-containing system, but still higher than that in neat buffer system and Tween-20-containing system. It is indicated that ChCl:Lys has good biocompatibility to the recombinant *E. coli* cells, and the cytotoxicity of substrate can be alleviated by introducing ChCl:Lys in the reaction system.

### Effects of various surfactants and NADESs on cell membrane permeability

Cell membrane permeability is related to the efficiency of mass transfer and whole-cell biotransformation (Talukder et al. 2019). To assess the effects of various surfactants and NADESs on the permeability of cell membrane, the OD_260_ and OD_280_ values were measured. As shown in Table 5, compared to the buffer system, the OD_260_ and OD_280_ values all increased in a surfactant-containing medium, implying that surfactants can enhance the permeability of cell membrane. Besides, with the addition of ChCl:Lys in the Tween-20-containing system, the OD_260_ and OD_280_ values were increased, but lower than that with Tween-20 only. It is likely that too much increase in cell membrane permeability may cause the cell death. Therefore, the enhancement of whole-cell catalytic efficiency may be attributed to the moderate increase in cell membrane permeability. To observe the changes of cell membrane more intuitively, the cells breeding in the Tween-20/ChCl:Lys-containing system were observed by SEM. As shown in Additional file 1: Fig. S4a, SEM images showed that the recombinant *E. coli* cells were integral in the neat PBS buffer solution. With the addition of Tween-20 in the reaction medium, the cell membrane forms small pores to intensify the mass-transfer efficiency (Additional file 1: Fig. S4b). Additional file 1: Fig. S4c depicted that the cell membrane became more permeabilized in the Tween-20/ChCl:Lys-containing system, which facilitates the mass transfer of substrate and biocatalyst, and thus accounted for the best yield obtained in this reaction system.

**Table 5:** The effects of various surfactants/NADESs on cell membrane permeability

Additives	OD_260_	OD_280_
PBS buffer	0.247 ± 0.02	0.205 ± 0.01
Tween-20	0.329 ± 0.02	0.276 ± 0.03
Tween-80	0.273 ± 0.04	0.235 ± 0.01
Span-20	0.266 ± 0.02	0.229 ± 0.04
Span-60	0.282 ± 0.04	0.232 ± 0.01
Span-80	0.295 ± 0.04	0.221 ± 0.02
ChCl:IPA (1:1)	0.247 ± 0.03	0.228 ± 0.03
ChCl:Gly (1:1)	0.302 ± 0.02	0.264 ± 0.04
ChCl:U (1:1)	0.269 ± 0.02	0.233 ± 0.04
ChCl:EG (1:1)	0.309 ± 0.04	0.271 ± 0.01
ChCl:Lys (1:1)	0.291 ± 0.03	0.262 ± 0.03
Tween-20 & ChCl:U (1:1)	0.294 ± 0.03	0.243 ± 0.04
Tween-20 & ChCl:Lys (1:1)	0.309 ± 0.02	0.257 ± 0.02
Tween-20 & ChCl:EG (1:1)	0.388 ± 0.03	0.291 ± 0.04

Reaction conditions: 17.2 g (DCW)/L recombinant *E. coli* cells, 0.6% (w/v) various surfactants, 4% (w/v) various NADESs, PBS buffer (pH 7.0), 30 ℃, 200 rpm for 18 h

### Scale-up preparation of (*R*)-MTF-PEL

The scale-up preparation of (*R*)-MTF-PEL in the developed Tween-20/ChCl:Lys-containing system was carried out under the optimal reductive conditions. 200 mM of 3'-(trifluoromethyl)acetophenone was added into a 500 mL Erlenmeyer flask, which containing 100 mL Tween-20/ChCl:Lys-buffer mixed reaction medium. Under the optimum reductive conditions, the yield achieved 91.0% after 18 h reaction, and the ee value was above 99.9%. It indicated that the developed bioprocess for the (*R*)-MTF-PEL preparation in the Tween-20/ChCl:Lys-containing system was also feasible on 500 mL shaken flask with 100 mL working volume.

## Discussion

As shown in Table 1, although the recombinant *E. coli* LXCAR-S154Y reduced 3'-(trifluoromethyl)acetophenone with ee > 99.9%, the product yield was only 26.9%. Thus, it was necessary to improve the catalytic system to boost the yield. It was reported that carbohydrates and alcohols as co-substrates can promote coenzyme regeneration, and play an important role in redox biocatalysis (Mourelle-Insua et al. 2019). In this study, some sugars and alcohols as co-substrates were assessed, and finally isopropanol exhibited the best promotion ability. When the amount of isopropanol was 15% (v/v), the yield reached 95.8% with > 99.9% ee under 50 mM substrate concentration (Fig. 1b). However, with further enhancement of isopropanol concentration, the catalytic efficiency decreased significantly, which may due to the toxic effect of excessive isopropanol on the cells (Sharma et al. 2017). Jiang et al. reported that isopropanol can promote coenzyme regeneration(Jiang and Fang 2020), and the production of coenzyme NADH was indeed observed by the supplement with isopropanol in the cell-free extract of the recombinant *E. coli* cells (Table 3). At the optimized reaction conditions, the product yield achieved 93.8% with ee > 99.9% under 100 mM substrate concentration (Fig. 2).

Although 93.8% yield was obtained at 100 mM substrate concentration (Fig. 2e), the increase of substrate concentration significantly reduced the conversion efficiency, so an increasing conversion efficiency was desirable. It has been reported that the introduction of DES as cosolvent can improve the substrate loading and obtain higher biocatalysis efficiency by improving the permeability of cellular membrane in the biotransformation (Xiong et al. 2021; He et al. 2020). Surfactants, as a kind of cosolvent to improve the solubility of hydrophobic compounds, are helpful in the biocatalysis process (Benvenutti et al. 2019; Patzold et al. 2019). Therefore, a dual cosolvent synergistic system with surfactant and NADES was constructed in this study. As shown in Additional file 1: Table S1, when Tween-20 was added into the buffer, the solubility of the substrate increased from 425.5 mg/L to 739.6 mg/L, which proved the solubilizing ability of surfactant. Moreover, when adding 0.6% (w/v) Tween-20, the product yield reached 82.6% under 200 mM substrate concentration, while a 74.1% yield in the buffer system (Fig. 3a, b). These results indicated that the addition of Tween-20 increases the solubility of the substrate and promotes the biotransformation efficiency.

In this work, we found that the ChCl-based NADESs achieved better catalytic efficiency than other NADES, and ChCl:Lys (1:1) performed best (Table 2 & Fig. 4a). With the addition of 4% (w/v) ChCl:Lys, the yield of 87.9% was acquired under 200 mM substrate concentration (Fig. 4b). In addition, the effect of ChCl:Lys (1:1) on the biotransformation was further investigated. It could be observed that the introduction of ChCl:Lys could moderately improve the solubility of 3'-(trifluoromethyl)acetophenone (Additional file 1: Table S1), but exhibited better biocompatibility towards cells and optimally increased the cell permeability (Table 5, Fig. 6 & Additional file 1: Fig. S4). Our experimental results also showed that the introduction of NADES promotes the coenzyme regeneration in the process of biocatalysis (Table 3). These data indicated that the construction of a dual co-solvent synergistic system is suitable for increasing the substrate loading in the process of biocatalysis. Furthermore, the established synergistic system containing surfactant/NADES dual co-solvent is also applicable to the bioreduction of ketoester and other aromatic ketones (Table 4).

In summary, we developed an effectively bioprocess for the (*R*)-MTF-PEL preparation with recombinant *E. coli* whole cells, affording carbonyl reductase gene from *Leifsonia xyli* HS0904. A synergistic system containing surfactant/NADES dual co-solvent was constructed, and consequently enhanced the whole-cell catalytic efficiency for (*R*)-MTF-PEL. After the parameter optimization for the bioreduction, (*R*)-MTF-PEL was produced in good yield and excellent enantiopurity in the constructed surfactant/NADES dual co-solvent synergistic system, which is the highest ever reported. The developed integration strategy of surfactant and NADES has great potential in the biocatalytic process and the synthesis of chiral alcohols.

## Supplementary Information

**Additional file 1: Fig. S1.** GC chromatogram; **Fig. S2.** The ^1^H NMR and ^13^C NMR spectra of the product; **Fig. S3.** The ^1^H NMR spectrum of ChCl:Lys (1:1); **Fig. S4.** SEM images of recombinant *E. coli* cells in different reaction media; **Figure S5.** The standard curve of 3'-(trifluoromethyl)acetophenone and (*R*)-MTF-PEL; **Table S1.** The solubility of 3'-(trifluoromethyl)acetophenone in different media; **Table S2.** Effect of different additives on the asymmetric reduction catalyzed by recombinant *E. coli* cell; **Table S3.** Effect of ChCl:Lys and its components on 3'-(trifluoromethyl)acetophenone bioreduction to (*R*)-MTF-PEL with recombinant *E. coli* cells.

## Data Availability

The datasets supporting the conclusions of this article are included within the article and its Additional file 1.

## Abbreviations

(*R*)-MTF-PEL: (*R*)-1-[3-(Trifluoromethyl)phenyl]ethanol
ee: Enantiomeric excess
ChCl:Lys: Choline chloride: lysine
(*S*)-CPMA: (*S*)-(4-Chlorophenyl)-(pyridin-2-yl)methanol
NADES: Natural deep eutectic solvent
HBA: Hydrogen bond acceptor
HBD: Hydrogen bond donor
ChCl:Bd: Choline chloride:1,4-butanediol
COBE: Ethyl 4-chloro-3-oxobutanoate
(*S*)-CHBE: (*S*)-4-Chloro-3-hydroxybutyrate
GC: Gas chromatography
MAR: Metabolic activity retention
SEM: Scanning electron microscope
CTAB: Cetyltrimethyl ammonium bromide
SDBS: Sodium dodecyl benzene sulfonate
CA: Cortisone acetate
