# Supplementary material for: Effective asymmetric preparation of (R)-1-[3-(trifluoromethyl)phenyl]ethanol with recombinant E. coli whole cells in an aqueous Tween-20/natural deep eutectic solvent solution
Source: AMB Express. 2021 Aug 19;11:118. doi: 10.1186/s13568-021-01278-6 (PMC8377109; doi:10.1186/s13568-021-01278-6)
Supplement: Supplementary file 1 — Additional file 1: Fig. S1. GC chromatogram; Fig. S2. The 1H NMR and 13C NMR spectra of the product; Fig. S3. The 1H NMR spectrum of ChCl:Lys (1:1); Fig. S4. SEM images of recombinant E. coli cells in different reaction media; Figure S5. The standard curve of 3'-(trifluoromethyl)acetophenone and (R)-MTF-PEL; Table S1. The solubility of 3'-(trifluoromethyl)acetophenone in different media; Table S2. Effect of different additives on the asymmetric reduction catalyzed by recombinant E. coli cell; Table S3. Effect of ChCl:Lys and its components on 3'-(trifluoromethyl)acetophenone bioreduction to (R)-MTF-PEL with recombinant E. coli cells. [file 13568_2021_1278_MOESM1_ESM.docx]

**AMB Express**

**Supporting Information:**

**Effective asymmetric preparation of** [**(*R*)-1-[3-(trifluoromethyl)phenyl]ethanol**](https://www.chemsrc.com/en/cas/127852-24-8_900872.html) **with recombinant *E. coli* whole cells in an aqueous** **Tween-20/natural deep eutectic solvent solution**

**Wenjin Zhuang^1^ · Hanyu Liu^1^ · Ying Zhang**^1^ **· JunYao He**^2^ **· Pu Wang**^1^
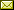


^1^ Key Laboratory of Green Pharmaceutical Technologies and Related Equipment of Ministry of Education, College of Pharmaceutical Science, Zhejiang University of Technology, Hangzhou 310014, China

^2^ Zhejiang Pharmaceutical College, Ningbo 315100, China

*Corresponding author:

Dr. Pu Wang

Professor

Key Laboratory of Green Pharmaceutical Technologies and Related Equipment of Ministry of Education, College of Pharmaceutical Science, Zhejiang University of Technology, Hangzhou 310014, China

E-mail: [wangpu@zjut.edu.cn](mailto:wangpu@zjut.edu.cn) (P. Wang)

Telephone and Fax: +86-571-88320389


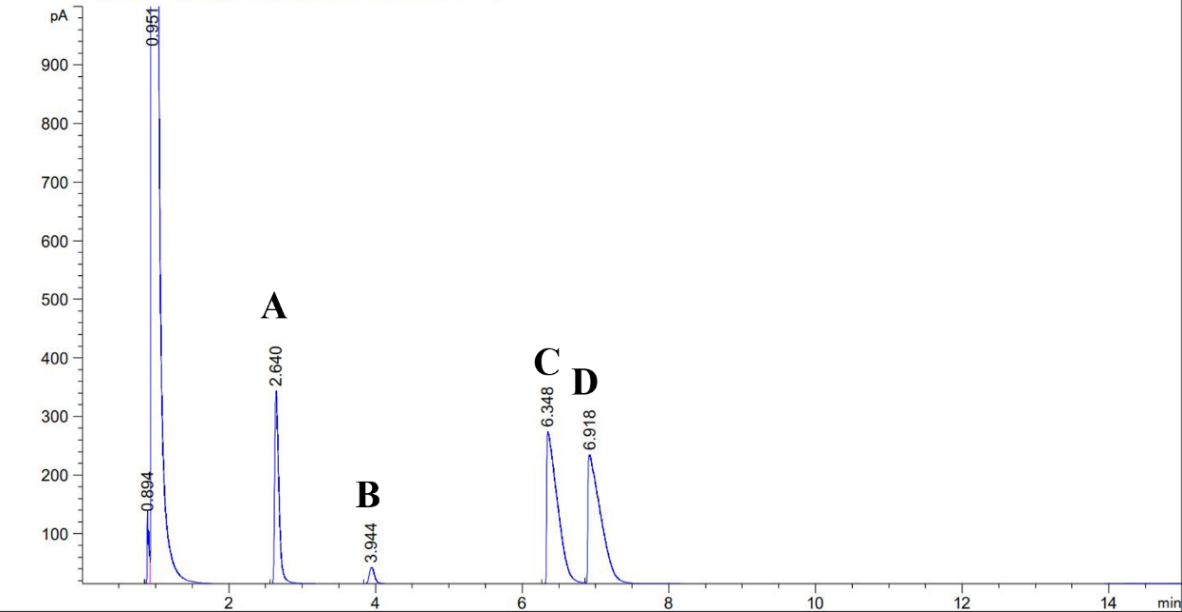


**Fig. S1 GC chromatogram**

A: 3'-(trifluoromethyl)acetophenone, B: *n*-dodecane, C: (*R*)-MTF-PEL, D: (*S*)-MTF-PEL

^1^H NMR (600 MHz, CDCl_3_) δ 7.81-7.36 (m, 4H), 5.12-4.85 (m, 1H), 1.99 (s, 1H), 1.66-1.36 (m, 3H).


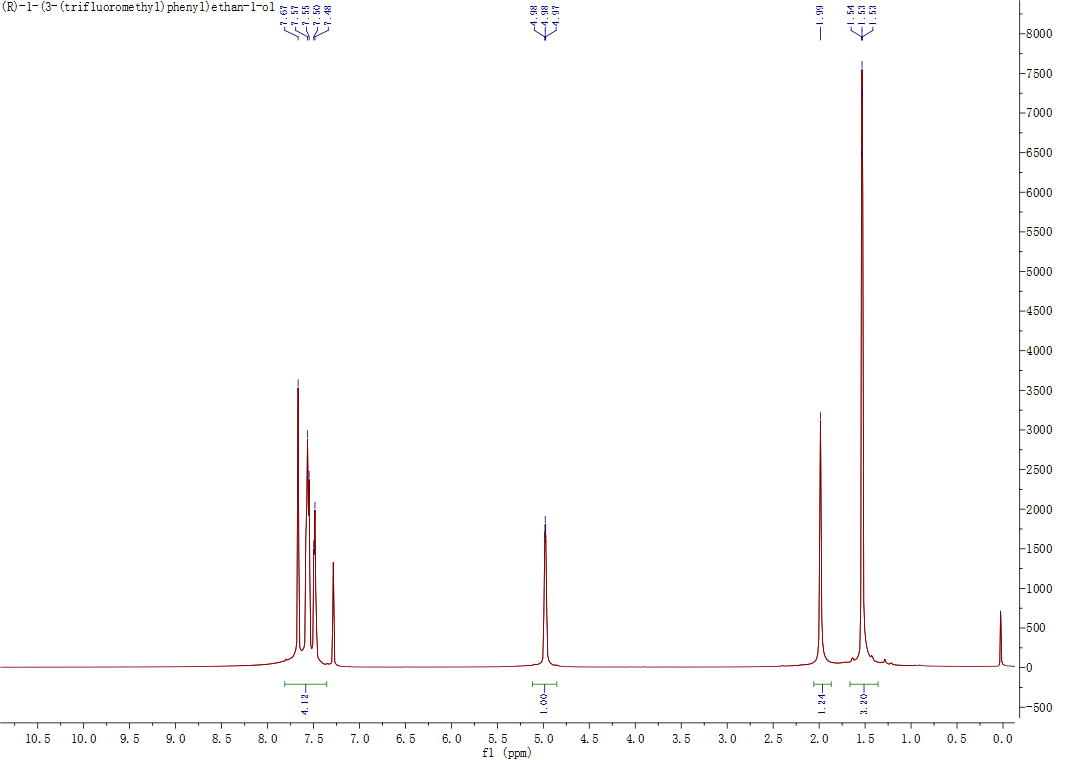


^13^C NMR (151 MHz, CDCl_3_) δ 146.71, 130.92, 130.71, 128.92, 128.76, 125.05, 124.23, 124.20, 123.25, 122.21, 122.18, 69.82, 25.35.


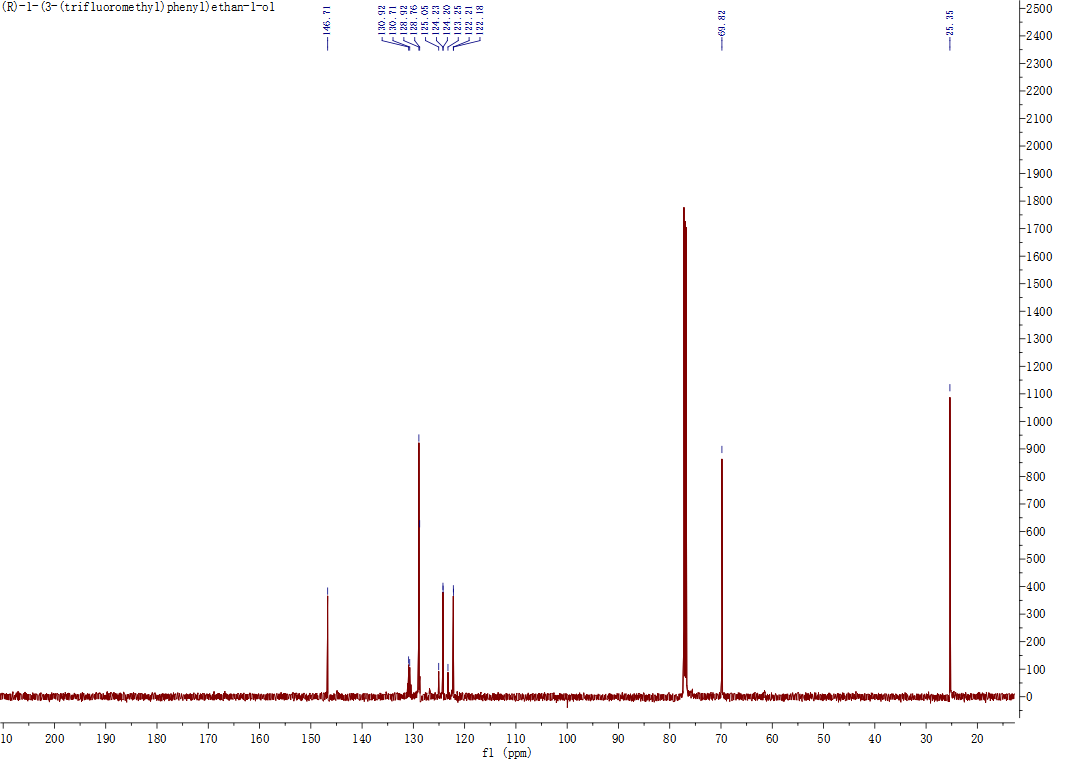


**Fig. S2 The ^1^H NMR and ^13^C NMR spectra of the product**

^1^H NMR (400 MHz, D_2_O) δ: 3.96-3.92 (m, 2H, -CH_2_OH), 3.42-3.39 (t, 2H, -CH_2_N), 3.33-3.30 (t, 1H, -CHNH_2_), 3.09 (s, 9H, -(CH_3_)3N), 2.88-2.84 (t, 2H, -CH_2_NH_2_),

1.66-1.52 (m, 4H, -CH_2_CH_2_), 1.36-1.24 (m, 2H, -CH_2_)


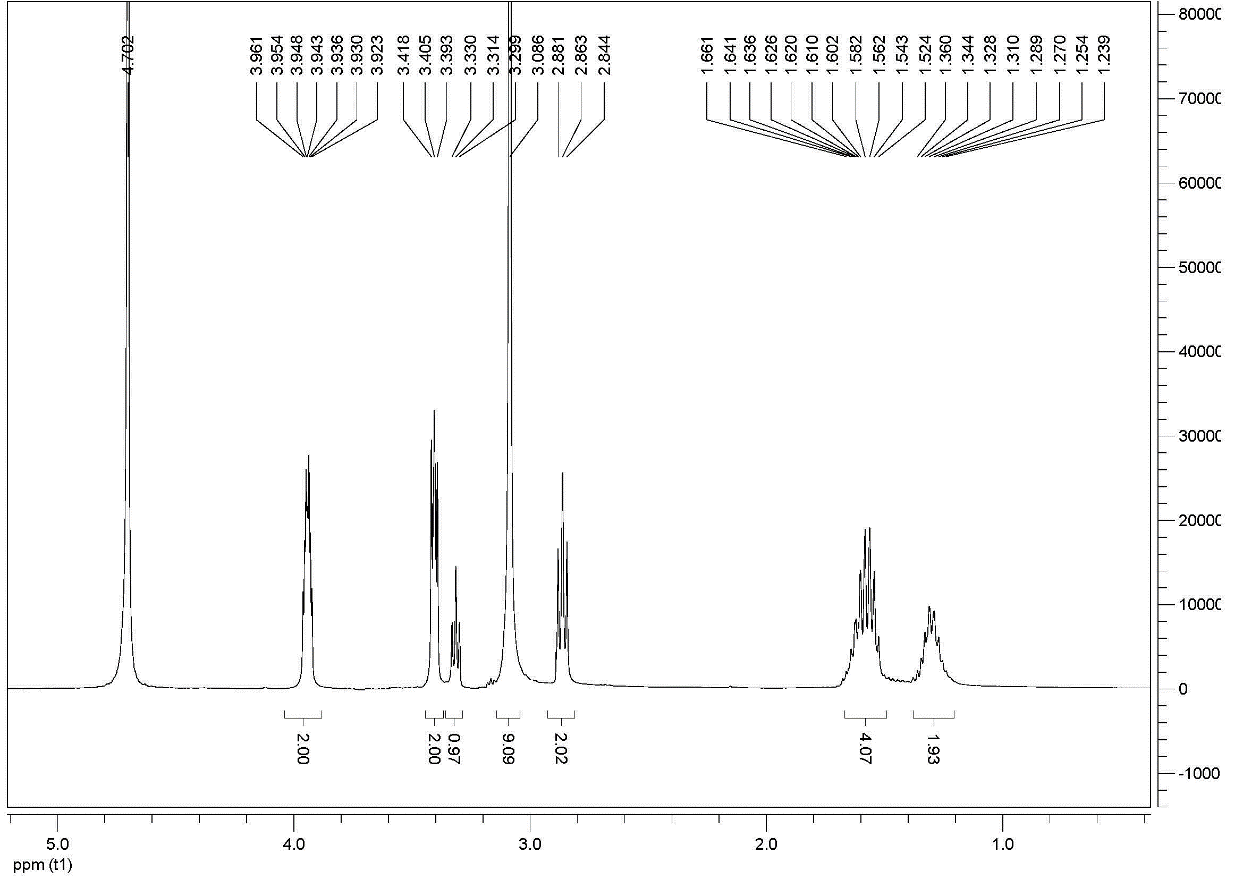


**Fig. S3 The ^1^H NMR** **spectrum of** **ChCl:Lys (1:1)**


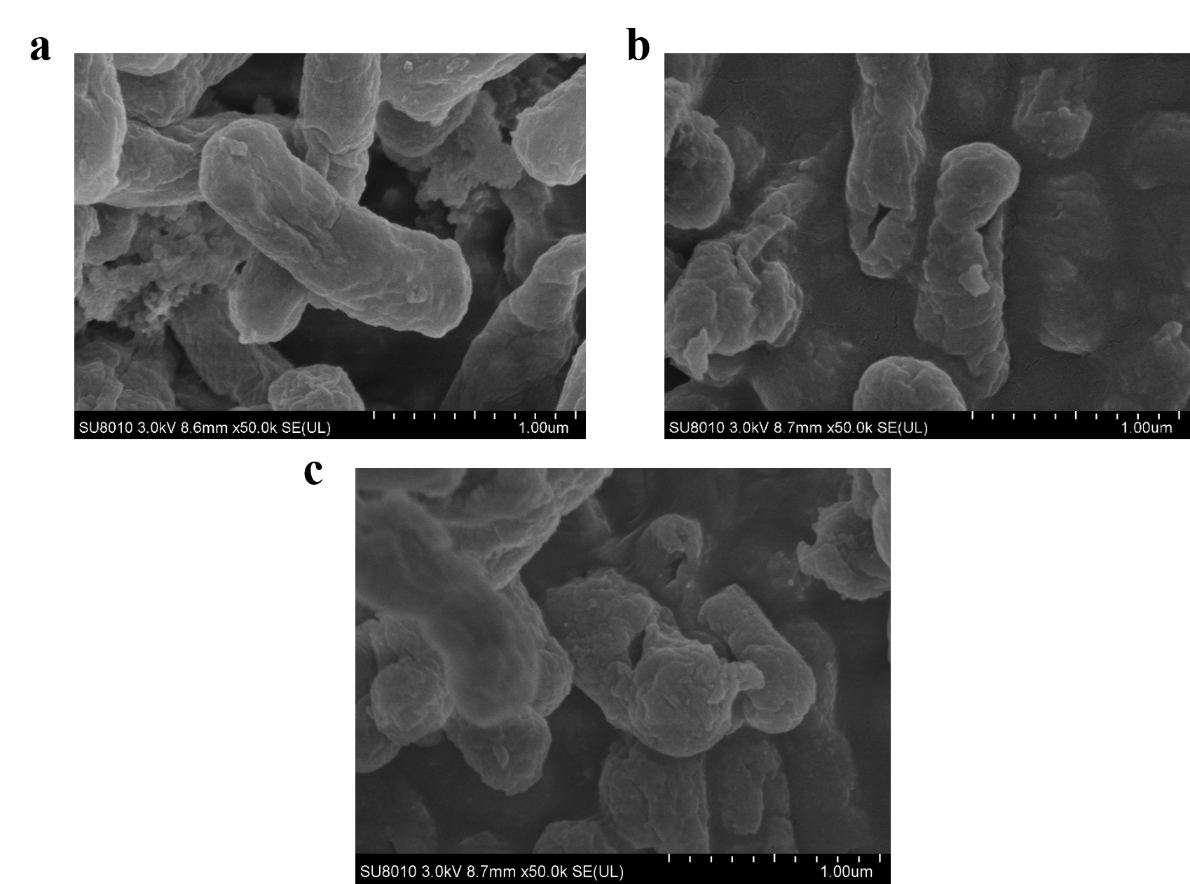


**Fig. S4 SEM images of recombinant *E. coli* cells in different reaction media**

**a.** Cells incubated in the PBS buffer reaction system for 18 h, 15% (v/v) isopropanol as co-substrate

**b.** Cells incubated in the Tween-20-containing reaction system for 18 h, 15% (v/v) isopropanol as co-substrate, 0.6% (w/v) Tween-20

**c.** Cells incubated in the Tween-20/ChCl:Lys-containing reaction system for 18 h, 15% (v/v) isopropanol as co-substrate, 0.6% (w/v) Tween-20, 4% (w/v) ChCl:Lys

Magnification of a, b, c was 50000 times.


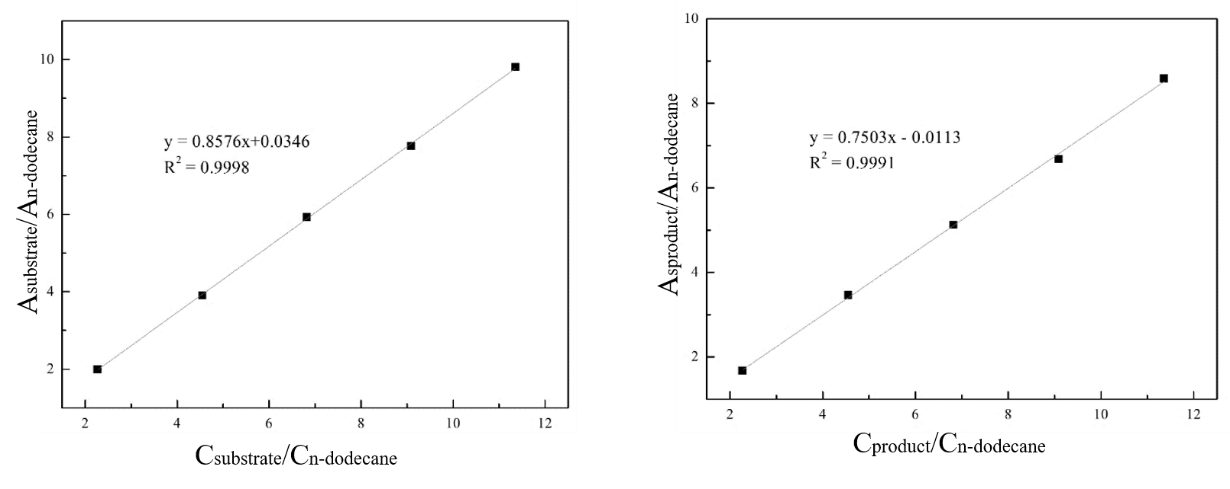


**Figure S5 The standard curve of 3'-(trifluoromethyl)acetophenone and (*R*)-MTF-PEL**

**Table S1** **The solubility of 3'-(trifluoromethyl)acetophenone in different media**

| Solvent | Solvent content | Solubility (mg/L) |
| --- | --- | --- |
| PBS buffer | -- | 425.5±1.87 |
| PBS buffer + isopropanol | 15% (v/v) | 437.6±1.55 |
| PBS + Tween-20 | 0.6% (w/v) | 739.6±1.21 |
| PBS + ChCl:Lys | 4% (w/v) | 513.9±2.58 |
| PBS + isopropanol &Tween-20 & ChCl:Lys | 15%(v/v)&0.6%(w/v)&4%(w/v) | 806.4±1.67 |

Reaction conditions: excess 3'-(trifluoromethyl)acetophenone was added in the PBS buffer (pH 7.5) with different solvents, 200 rpm, 30 ℃, shaken for 21 h

**Table S2 Effect of different additives on the asymmetric reduction catalyzed**

**by recombinant *E. coli* cells**

| Additives | Yield (%) | ee (%) |
| --- | --- | --- |
| -- | 74.1 | >99.9 |
| ^a^Tween-20 | 82.6 | >99.9 |
| ^b^ChCl:Lys (1:1) | 83.1 | >99.9 |
| ^a^Tween-20 & ^b^ChCl:Lys (1:1) | 89.2 | >99.9 |

Reaction conditions: 200 mM 3'-(trifluoromethyl)acetophenone, 17.2 g (DCW)/L recombinant *E. coli* cells, 15% (v/v) isopropanol, PBS buffer (pH 7.5), 30 ℃, 200 rpm, reaction for 21 h

^a^0.6% (w/v) Tween-20

^b^4% (w/v) ChCl:Lys

**Table S3 Effect of ChCl:Lys and its components on the asymmetric bioreduction with recombinant *E. coli* cells**

| Additives | Yield (%) | ee (%) |
| --- | --- | --- |
| -- | 74.1 | >99.9 |
| ^a^ChCl:Lys (1:1) | 85.6 | >99.9 |
| ^b^ChCl | 77.9 | >99.9 |
| ^b^Lysine | 79.6 | >99.9 |
| ^c^ChCl + lysine | 80.8 | >99.9 |

Reaction conditions: 200 mM of 3'-(trifluoromethyl)acetophenone, 17.2 g (DCW)/L recombinant *E. coli* cells, 15% (v/v) isopropanol, 4% (w/v) ChCl:Lys, PBS buffer (pH 7.5), 30 ℃, 200 rpm, reaction for 21 h

^a^4% (w/v) ChCl:Lys (1:1)

^b^ChCl and lysine were added on the basis of 4% (w/v) ChCl:Lys (1:1) content

^c^The mixture of individual components (ChCl + lysine) on the basis of 4% (w/v) ChCl:Lys (1:1) content
